# Heating by Gas

**Published:** 1914-05-16

**Authors:** Percy Wilde

**Affiliations:** 23 The Circus, Bath


### Heating by Gas.

*To the Editor of* The Hospital.

Sir,—I have seen in The Hospital a very kind appreciation of the "Altheat Stove." As its inventor I feel deeply grateful. May I explain why? Some twenty years ago I conceived the idea of a hospital which would admit *all* classes of patients and which would make no appeal to the public for subscriptions. I hate "subscribers."

Nobody believed in my idea except one present, and with her help I started the Lansdown Hospital at Bath. It has grown up. We have now thirty-five private wards in addition to the public ward for the poor. We have never appealed to the public for a single penny, and we are in a sound financial position. The question of heating the many wards has been always in my mind. I have expended many hundreds of pounds upon experiments—at last I brought the cost of heating to ¼d. per hour.

My friends, who were directors of gas companies, said that this was just what they wanted, but when I was able to produce the stove I found the opposition of the sales managers of the gas companies positively iniquitous. The fact that it only burned 10 feet of gas to heat a room, which *ought* to require 30 to 40 cubic feet of gas, damned it utterly. All our patients are delighted with the stove. What we want is a smaller one for the smaller wards. We want a large number of such stoves. Many of my friends also want them, but the company is handicapped by insufficient capital to produce them. It is rather funny, that starting with an effort to run a hospital without subscribers I should now find myself mostly concerned with the finances of a gas stove!

Should you ever be in Bath I shall be delighted to show you the Lansdown Hospital, of which I am very proud.—Believe me, very truly yours, Percy Wilde, M.D.

23 The Circus, Bath, April 22, 1914.

